# Hexokinase 2 (HK2), the tumor promoter in glioma, is downregulated by miR-218/Bmi1 pathway

**DOI:** 10.1371/journal.pone.0189353

**Published:** 2017-12-08

**Authors:** Hui Liu, Nan Liu, Yingduan Cheng, Weilin Jin, Pengxing Zhang, Xin Wang, Hongwei Yang, Xiaoshan Xu, Zhen Wang, Yanyang Tu

**Affiliations:** 1 Department of Experimental Surgery, Tangdu Hospital, Fourth Military Medical University, Xi’an, Shaanxi, China; 2 Department of Research, Cipher Ground, North Brunswick, New Jersey, United States of America; 3 School of Life Sciences and Biotechnology, Shanghai Jiao Tong University, Minhang, Shanghai, China; 4 Department of Bio-Nano-Science and Engineering, Institute of Micro-Nano Science and Technology, Shanghai Jiao Tong University, Minhang, Shanghai, China; 5 Department of Neurosurgery, Brigham and Women’s Hospital, Harvard Medical School, Boston, Massachusetts, United States of America; University of South Alabama Mitchell Cancer Institute, UNITED STATES

## Abstract

In cancer, glycolysis driving enzymes and their regulating microRNAs are one of the key focus of oncology research lately. The glycolytic enzyme hexokinase 2 (HK2) is crucial for the Warburg effect in human glioma, the most common malignant brain tumor. In the present study, we studied the tumorigenic role of HK2 in glioma, and clarified the mechanism of miR-218 induced HK2 regulation in glioma development. The HK2 expression in patient derived glioma and non neoplastic brain tissue was quantified. The HK2 silenced U87 and U251 cell lines were assessed for their proliferation, migration and invasive potential *in vitro*, while the tumor forming potential of U87 cells was evaluated *in vivo*. The untreated cell lines served as control. The HK2 expression in (a) lentivirus-infected, miR-218 overexpressing and (b) shRNA mediated Bmi1 silenced U87 and U251 glioma cell lines were quantified. Luciferase reporter assay, qRT-PCR analysis and WB were employed as required. The HK2 expression was significantly increased in glioma tissues comparing with the non neoplastic brain tissues and was positively correlated with the glioma grade. Silencing HK2 in glioma cell lines significantly decreased their proliferation, migration, invasion and tumorigenic abilities. Although, overexpression of miR-218 significantly downregulated the HK2 expression, luciferase reporter assay failed to show HK2 as the direct target of miR-218. A direct correlation, however, was observed between HK2 and Bmi-1, the direct target of miR-218. Taken together, our findings confirmed the tumorigenic activity of HK2 in glioma, and the involvement of the miR218/Bmi1 pathway in the regulation of its expression.

## Introduction

Glioma was the most common primary tumor of the central nervous system, which was characterized by aggressive proliferation, migration and invasion abilities. Despite the combined therapeutic approach, involving surgery, radiotherapy and chemotherapy, the median survival of most glioma patients was as low as one to two years, with the 5 year survival rate staying below 10% [1–2]. Angiogenesis was one of the hallmark malignancy parameter of glioma. Interestingly, despite its success in reducing blood flow, a preclinical assessment of anti-VEGF treatment revealed to increase the invasiveness of glioblastoma cells, the reason for which was identified as the activation of the glycolytic pathway [3–4]. In addition to this anerobic glycolysis, aerobic glycolysis, an unique metabolic phenomenon that convert glucose into lactic acid even in the presence of oxygen, referred to as the Warburg Effect, was noted in most solid tumors [5–7]. When compared to normal cells, tumor cells preferentially utilize this far less efficient process for ATP production, which also increased the cell’s proliferation, invasiveness and apoptosis resistance [8–9]. This high rate of glycolysis in tumor cells, including gioma cells, was presumably ascribed to up-regulation of key catalytic enzymes in glycolysis, especially hexokinases, more specifically hexokinase 2 (HK2) [10–11]. Indeed, elevated levels of HK2 had been found in many human tumors, localized to the outer membrane of mitochondria by binding the voltage-dependent anion channel (VDAC) transporter and thus had preferential access to mitochondrial ATP [12–14]. In addition to its critical metabolic role, HK2 could also promote glioma survival, against chemo or radiation insult, by repressing mitochondria mediated apoptotic pathway in glioma cells [15]. Thus, HK2 was of great interest in recent years and efforts were being made in understanding the associated underlying molecular mechanisms in glioma, towards which the present work was also dedicated.

There was much evidence suggesting that microRNAs (miRNAs), which could regulate genes by antisense complementarity to specific mRNAs, participated in diverse physiological and pathological activities in humans [16–19]. In addition to their direct oncogenic or tumor suppressor functions, miRNAs could also downregulate multiple target genes related to tumorigenesis and progression [20–21]. Previously we revealed the tumor suppressor function of miR-218, which could dramatically reduce the proliferation, migration, invasion and self-renewal of glioma cells, by targeting its functional downstream target Bmi1, a stem cell-promoting oncogene [22]. In response to increased expression of miR-218 in glioma cells, the gene chip microarray analysis revealed a decrease in their HK2 expression, which was further identified as a target of miR-218 by Target Scan. This indicated an association of miR-218 and HK2, which might play an important role in glioma. Consequently, we aimed to reveal the role of HK2 in tumorigenesis and development of glioma, and further explored the association between miR-218 and HK2.

## Materials and methods

### Patients and tissue samples

This study was approved by the Research Ethics Committee of Tangdu Hospital. Between 2011 and 2013, twenty-one glioma tissue samples and two non-neoplastic brain samples were obtained from the surgeries performed at the Neurosurgery Department of Tangdu Hospital, Fourth Military Medical University. None of the patients had received chemotherapy or radiotherapy prior to surgery. Written informed consent was obtained from all the patients.

### Immunohistochemistry and hematoxylin and eosin staining

The formalin-fixed, paraffin-embedded tissue samples, sectioned at 5 μm thickness, were immunostained using the labeled Streptavidin Biotin 2 System. After being baked at 60°C for 30 minutes, sections of glioma were dewaxed in xylene and hydrated in a series of decreasing concentrations of ethanol solutions. Heat mediated antigen retrieval was performed by heating the sections in citrate buffer (pH 6.0, Sigma-Aldrich, USA) for 20 minutes in a microwave oven. Following peroxidase blocking with 0.3% H_2_O_2_/methanol for 10 minutes, the sections were blocked with phosphate-buffered saline (PBS) containing 5% normal horse serum (Vector Laboratories Inc., Burlingame, CA, USA). Subsequent to being incubated at 4°C overnight with Anti-HK2 mouse monoclonal antibody (1:500, Abcam, Cambridge, MA, USA), the sections were incubated at room temperature with the Goat Anti-Mouse IgG-HRP antibody and avidin–biotin peroxidase (Vector Laboratories Inc., Burlingame, CA, USA). Labelling was achieved with diaminobenzidine solution (Zhongshan Golden Bridge, Beijing, China) and counterstained with Meyer’s hematoxylin (Sigma Chemical Co., St Louis, MO, USA). Non-neoplastic brain tissues and non-immune IgG served as negative controls. Following standard procedure, the samples were also stained with hematoxylin and eosin (H&E). The stained sections were photographed using an optical or confocal microscope (Olympus). All samples were classified according to the fourth edition of the “histological grades of tumors of the central nervous system” published by the WHO in 2007.

IHC scoring: The percentage of positive cells, immunoreactive to HK2 antibody, was calculated from ten representative microscopic fields per sample, and scored as follows: 0 (0%), 1 (1%-10%), 2 (11%-50%) and 3 (>50%). The staining intensity was visually scored and stratified as follows: 0 (negative), 1 (weak), 2 (moderate) and 3 (strong). The final immunoreactivity score (IRS) was obtained for each case by multiplying the percentage of positive cells and the staining intensity score.

### Cell lines and cell culture

U87, U251 and HEK-293T cells were purchased from the Chinese Academy of Sciences Cell Bank. All cell lines were cultured in Dulbecco's Modified Eagle Medium (DMEM) supplemented with 10% FBS, 2 mM glutamine, 100 units of penicillin/ml, 100 ng of streptomycin/ml, and incubated at 37°C with 5% CO_2_.

### Plasmids and transfection

Plasmids containing pGPU6/GFP/Neo vector and three independent short hairpin RNAs (shRNAs) for reducing HK2 expression were purchased from GenePharma (Shanghai, China), as was the empty vector control. U87 and U251 were seeded onto six-well plates and pre-cultured to about 60% confluence before transfection. Plasmid transfection into cells was achieved using Lipofectamine 2000 (Invitrogen), following manufacturer’s instructions. Two days after transfection, cells were harvested for further analysis. For selection of glioma stable cell lines, U87 and U251 cells were cultured in 400 mg/mL neomycin (G418) for 14 days after transfection. The expression of HK2 was confirmed by quantitative reverse transcription PCR (qRT-PCR) and Western blotting.

### Lentivirus packaging and establishment of stable cell lines

A lentiviral packaging kit was purchased from Open Biosystems. Lentivirus carrying hsa-miR-218 or hsa-miR-negative control (miR-NC) and Bmi1 shRNA or its negative control (shNC) was packaged following the manufacturer's manual. HEK-293T cells were transfected with the lentivirus and the viral concentrate was obtained from the medium supernatant. Stable cell lines were established by infecting lentivirus into U87 and U251 cells in the presence of 8 μg /ml polybrene, followed by their selection in the presence of puromycin (2 μg /ml), for 14 days. The expression of miR-218 and HK2 were confirmed by qRT-PCR and Western blotting.

### Cell proliferation assay

Cell viability was analyzed using a Cell Counting Kit-8(CCK-8) (Sangon Biotech, China). Briefly, at a seeding density of 20,000 cells/ml of medium, stably-transfected cells/well were seeded in 96-well plates. At the 0 h, 24 h, 48h and 72h time points, the cells were incubated with CCK-8 solution at 37°C for 2h, before subjecting them spectroscopic analysis. The optical density (OD) value was determined as 450 nm using a microplate reader (Infinite M200). The experiment was done in triplicate and the average of the OD450 values was used to calculate cell growth rate.

### Wound healing assays

Stably-transfected cells were cultured in monolayer to 95% confluence, in six-well plates. The cell layer was scratched using a 200-mL pipette tip to create wound gaps and washed twice with PBS to remove the floating cells. The cells were then incubated in the serum free DMEM and cell migration into the wound was observed at three predetermined time points, 0, 12, and 24 hours, in randomly selected microscopic fields for each condition and time point. Images were acquired with a light microscope (Olympus, Tokyo, Japan) at 100× magnification. The distance traveled by the cells was determined by measuring the change in the wound width at different time points, compared to that at time 0h. The values obtained were expressed as a migration percentage, setting the width of the wound at 0 hour as 0%.

### Cell invasion assay

The top side of polycarbonate Transwell filter, in the top chamber of the QCM 24-Well Cell Invasion Assay (Cell Biolabs, Inc.), was coated with Matrigel. Cells (5X10^5^) were then seeded over the Matrigel under serum free medium, while the medium supplemented with serum served as a chemoattractant in the bottom chamber. The cells were incubated at 37°C for 48 h. The noninvasive cells in the top surface of Transwell filter were removed using cotton swabs. The cells which successfully invaded on to the lower surface of the Transwell filter were fixed in 100% methanol for 15 minutes, air-dried and stained with 0.1% crystal violet for 10 minutes, prior to counting under a microscope (five random fields of view at 100× magnification). The data was presented as the mean of three independent experiments.

### Tumorigenic assay

For tumorigenesis assays, HK2-silenced U87 stable cells and their corresponding negative controls were implanted in the left and right flanks, respectively, of 4-week-old female NOD/SCID mice (3.0×10^6^/200 mL per mice, 6 mice per cell line), which were purchased from Animal Center of Fourth Military Medical University. Starting from day 1, the next day after cell implantation, tumor volumes were determined by measuring its length (a) and width (b) every other day, up to 28 days. The tumor volume (V) was calculated according to the formula V = (ab)^2^/2. After the mice were sacrificed, tumors were formalin-fixed, paraffin-embedded, and sectioned for IHC and H&E staining. Experiments were carried out in accordance with institutional guidelines and regulations of the government (S1 File).

### Luciferase reporter assay

The luciferase reporter assay was performed to assess if miR-218 alters HK2 expression by post-transcriptional regulation on the 3’UTR of the HK2 gene. The Hsa-miR-218 vector (GenePharma Co.) and luciferase reporter plasmids, containing putative wild-type (pmirGLO-HK2 3’-UTR) and mutant (pmirGLO-HK2 3’-UTR-mut) 3’UTR binding sites of HK2, were cotransfected into HEK293FT cell line. Cell lysates were prepared at 48 hours post-transfection. The luciferase activity was measured using the Dual-Luciferase Reporter Assay System (Promega), and the results were normalized to those of the control group. All reactions were repeated in triplicate through independent experiments.

### Quantitative real-time PCR

Total RNA was extracted from cells by using TRIzol reagent (Invitrogen, Carlsbad, CA, USA), and then reverse transcribed into cDNA using PrimeScript RT Reagent Kit (Takara, Dalian, China) by following the manufacturer’s protocol. Realtime PCR assay was performed on an ABI 7500 real-time PCR system (Applied Biosystems, Foster City, CA, USA) using the SYBR PrimeScript RT-PCR kit (Takara) with specific primers. GAPDH was used as an internal control and fold changes were calculated by relative quantification (2^-ΔΔCt^). All reactions were repeated in triplicate through independent experiments.

### Western blotting

The total cell lysates were prepared in high KCl lysis buffer (10 mmol/L Tris–HCl, pH 8.0, 140 mmol/L NaCl, 300 mmol/L KCl, 1 mmol/L EDTA, 0.5% Triton X-100, and 0.5% sodium deoxycholate) with complete protease inhibitor cocktail (Roche). The protein concentration was determined using a BCA Protein Assay Kit (Pierce). The samples were resolved in 10% SDS-PAGE gels and transferred to polyvinylidene fluoride (PVDF) membranes. Immunoblots were incubated with primary antibodies (HK2—1:500, Bmi1—1:1000, and GAPDH—1:1000; Abcam, Cambridge, MA, USA) overnight at 4°C. All primary antibodies were used according to the manufacturer’s instructions. After that, the membrane was incubated with the corresponding HRP linked anti-rabbit/mouse IgG antibody (1:5000, Zhongshan Golden Bridge, China) at room temperature for 2 h. The blots were visualized using the ECL-Plus reagent (Millipore, Billerica, MA, USA).

### Statistical analysis

Data were expressed as the means ± standard error mean (SEM) from three independent experiments. Two independent sample t-tests were performed using GraphPad Prism 5.0 software in order to detect significant differences in measured variables among groups. P value < 0.05 was considered to be statistically significant.

## Results

A total of 16 male and 5 female glioma patients, median age of 45.3 years (range 31–78), were enrolled in this study. None of the patients had received chemotherapy or radiotherapy prior to surgery. The clinicopathological features of all of the patients are listed in Table 1. Paraffin-embedded sections of non-neoplastic brain tissues were also included as controls. Nine of the 21 gliomas were classified as low-grade gliomas [3 pilocytic astrocytomas (WHO I) and d6 diffuse astrocytomas (WHO II)], and 12 were classified as high-grade gliomas [6 anaplasia astrocytomas (WHO III) and 6 primary glioblastomas (WHOIV)].

**Table 1:** Clinicopathological features of 21 patients with glioma.

Features	WHO I	WHO II	WHO III	WHO IV	Total
**Case No**.	3	6	6	6	21
**Gender**					
**Male**	1	5	6	4	16
**Female**	2	1	0	2	5
**Age**					
**<55**	3	5	6	4	18
**>55**	0	1	0	2	3
**Mean Age**	42.7	47.5	43.7	47.3	45.3

### An increased expression of HK2 in glioma tissues

**Fig 1:**
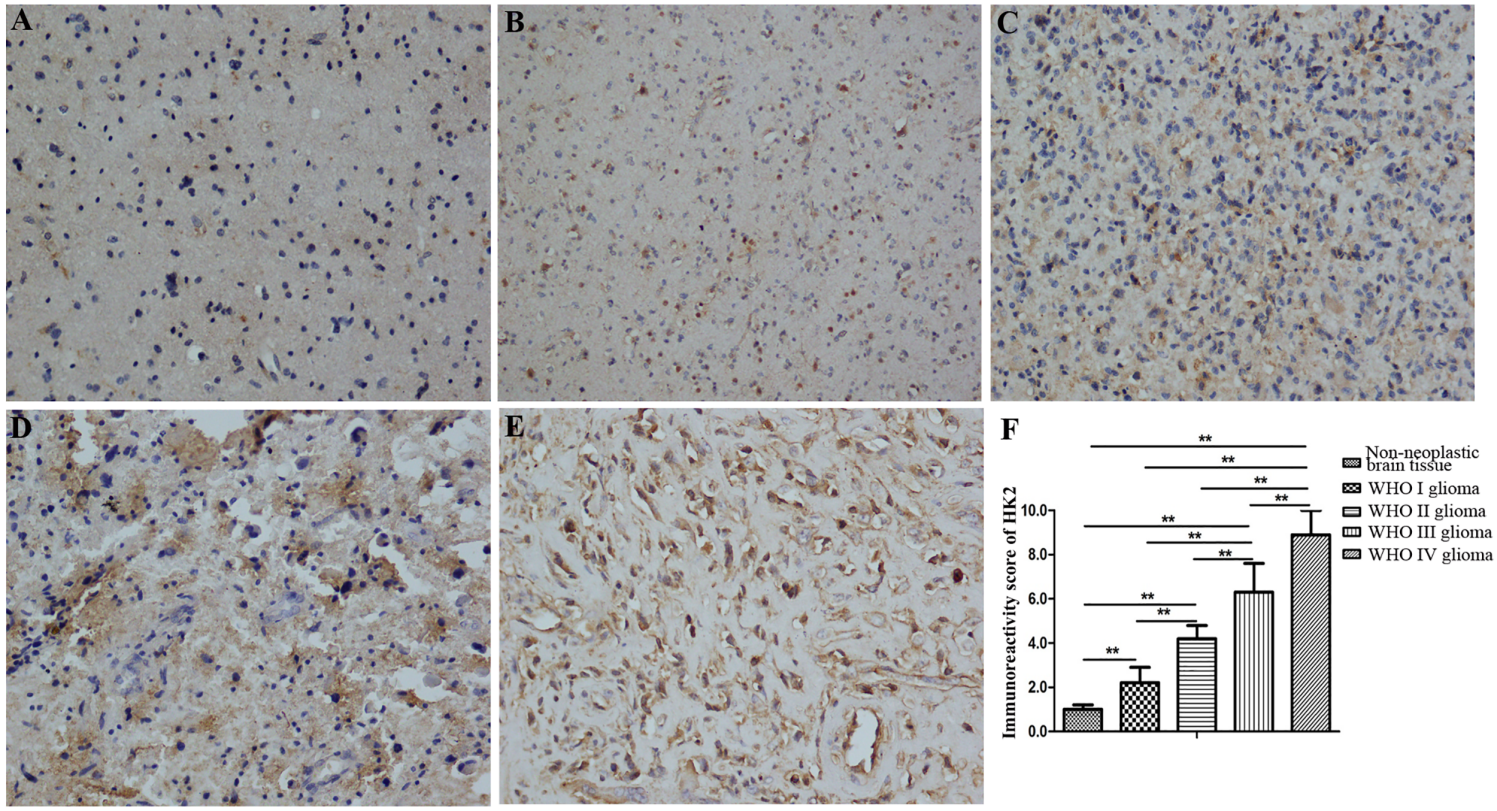
Immunohistochemistry to detect the expression of HK2 in glioma tissues. HK2 localized mainly to the cytoplasm. The expression of HK2 in different grades of glioma samples: (A). Non-neoplastic brain tissue; (B). Pilocytic astrocytomas (WHO I); (C). Diffuse astrocytomas (WHO II); (D). Anaplasia astrocytomas (WHO III); (E). Primary glioblastomas (WHO IV). Original magnification, 200×. (F). The immunoreactive score demonstrating a significant positive correlation between HK2 expression and glioma grade. Error bars represent SEM. **, P<0.01.

We detected the HK2 expression in glioma and non-neoplastic brain tissue samples by IHC. HK2 was mainly distributed in the cells, localized in cytoplasm. The IRS revealed a significant increase in HK2 expression in glioma samples compared to non-neoplastic brain tissue (Fig 1F). Further, amongst the glioma samples, HK2 expression increased significantly with the progression in tumor grade, thus establishing a positive correlation (Fig 1F, S1 Table). The patient’s age and gender factors failed to influence HK2 expression in glioma.

### Silencing HK2

**Fig 2:**
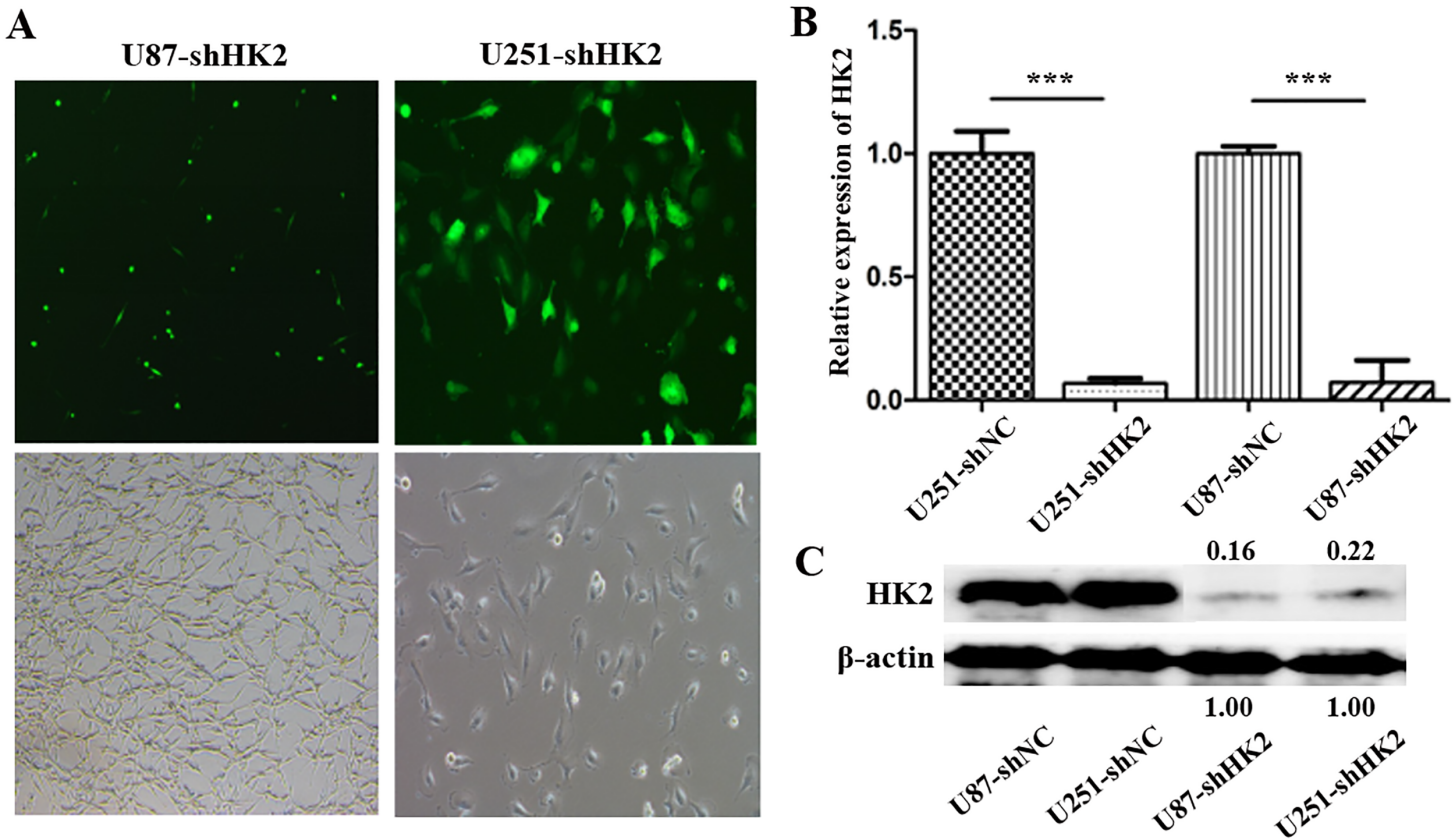
Silencing HK2 expression of glioma cells. (A). Successfully established HK2 silenced stable U87 and U251 cells (B). qRT-PCR analysis demonstrated the downregulation of HK2 expression in transfected U87 and U251 cells. (C). Western-blot analysis demonstrating the downregulation of HK2 protein in transfected U87 and U251 cells. Error bars represent SEM. ***, P<0.001.

To assess the role of HK2 in glioma, the enzyme was suppressed using short-hairpin RNA (shRNA) in U87 and U251 glioma cell lines. Post transfection, neomycin (G418) mediated stable U87 and U251 cells were obtained (Fig 2A), the analysis (qRT-PCR and western-blot) of which demonstrated a successful inhibition of HK2. There was significantly decreased HK2 RNA and protein expression after the inhibition (Fig 2B and 2C).

### Downregulation of HK2 inhibited glioma cell proliferation, migration, and invasion

**Fig 3:**
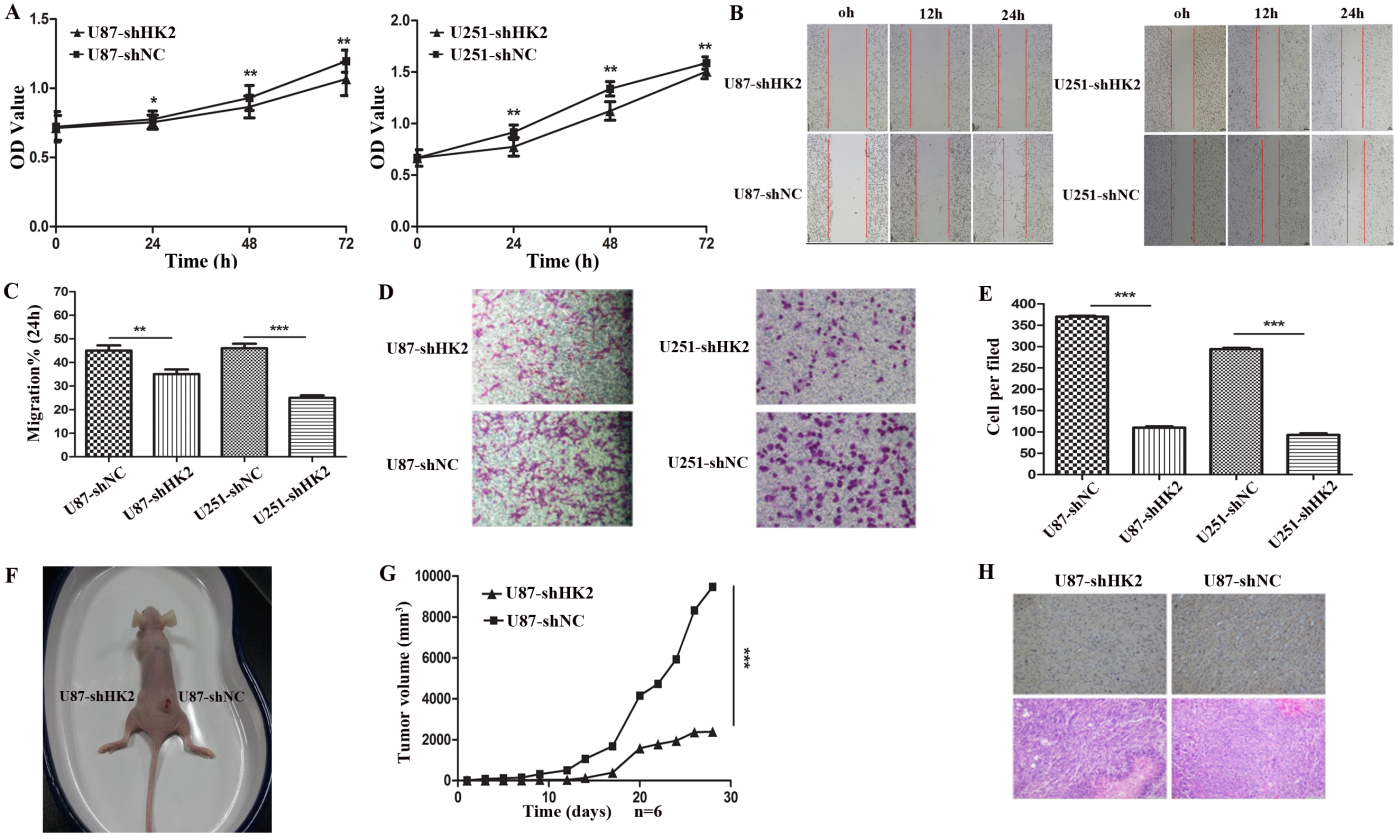
Silencing HK2 inhibits glioma cell invasion, migration, and proliferation *in vitro* and tumorigenesis *in vivo*. (A). Growth curves after cell transfections were assessed by CCK-8 assay. The rate of cellular proliferation was significantly decreased in both the HK2-silenced cell lines (B-C). The wound healing assay showed a significant difference in cell migration between transfected U87/U251 cells and their corresponding control. (B). Representative images taken at different time points. (C). Quantification of cell motility by measuring the wound width. The amount of motility was expressed as a migration percentage, setting the width of the wound at 0 hour as 0%. The rate of cellular migration was significantly decreased in both the HK2-silenced cell lines. (D-E). Transwell assay of transfected glioma cells. (D). Representative fields of invaded cells on the membrane. (E). Quantitative analysis of the invasive cells from three independent experiments. The rate of cellular invasion was significantly decreased in both the HK2-silenced cell lines. (F). In-vivo tumorigenesis; Nude mice were subcutaneously injected with HK2 silenced stable U87 cells and their corresponding negative controls (seeding density = 3.0×10^6^). (G). Determination of the tumor growth. Tumor volume was calculated every two days after injection, demonstrating a significant (p = 0.011) decrease in the tumor volume in HK2 silenced stable U87 cells. The mean volumes of xenograft tumors generated from HK2-silenced U87 cells were significantly smaller than those originating from its negative control cells. (H). IHC and H&E staining of xenograft tumor tissues. Magnification, 100×. Error bars represent SEM, *, P<0.05; **, P<0.01; ***, P<0.001.

The obtained HK2 silenced, stable U87 and U251 cells were assessed for their proliferation, migration and invasive abilities. As revealed by CCK-8 assay, the rate of cellular proliferation was significantly decreased in both the HK2-silenced cell lines (P<0.05), compared to their respective negative controls, at all the selected time points (Fig 3A). In a wound healing model, the HK2-silenced cells exhibited a considerably slower migration compared to their corresponding negative controls. The 24-hour wound closure rate was quantified as 35% and 25% for HK2-silenced U87 and U251 cells, respectively, while it was 45% and 46% for their corresponding U87 and U251 negative control cells, respectively (Fig 3B and 3C). Furthermore, transwell invasion assay demonstrated a reduced invasive potential of HK2-silenced glioma cells, to approximately one-third of their corresponding negative control cells (Fig 3D and 3E).

### Tumorigenic assay

The tumorigenic role of HK2 in glioma cells was assessed *in vivo*, using nude mice. At 20 days post-implantation, and from then on, the mean volumes of xenograft tumors generated from HK2-silenced U87 cells were significantly smaller than those originating from its negative control cells (p = 0.011; Fig 3F and 3G, S2 Table). A decrease in cell density and HK2 expression was evident in the HK2-silenced U87 xenografts, compared to its negative control tumors, as revealed through H&E and IHC staining (Fig 3H).

### The negative correlation between miR-218 and HK2 expression in glioma cells

**Fig 4:**
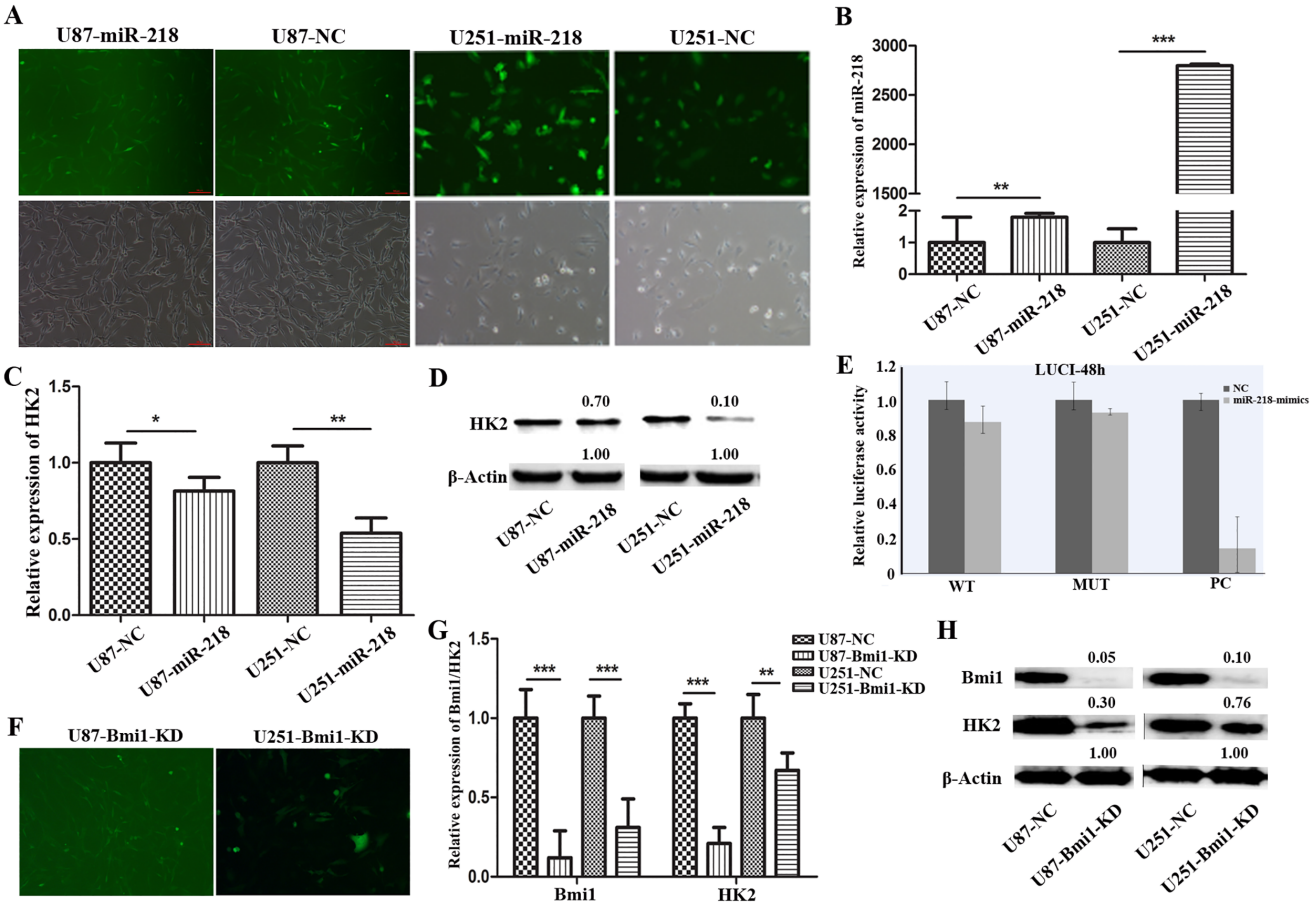
HK2 levels are inversely correlated with miR-218 levels in glioma cells, but HK2 is not a direct target of miR-218; miR-218 may regulate HK2 through Bmi1. (A). Establishment of stable LV-infected U87 and U251 cells overexpressing miR-218. (B). qRT-PCR analysis confirmed the increased miR-218 expression in glioma cell lines. (C). Overexpression of miR-218 down-regulated the mRNA level of HK2. (D). Overexpression of miR-218 down-regulated the protein level of HK2. (E). Luciferase reporter assay showed HK2 was not the direct target of miR-218. (F). Establishment of Bmi1 knocked-down stable U87 and U251 cells. (G). Knocking-down Bmi1 decreased the HK2 mRNA level. (H). Knocking-down Bmi1 also decreased the HK2 protein level.

We previously demonstrated the robust tumor suppressor role of miR-218 in glioma cells, the overexpression of which also showed, through gene chip analysis, a negative effect on HK2 expression, which was further analyzed here. The stable U87 and U251 cell lines overexpressing miR-218 were generated through lentivirus infection and puromycin mediated selection, and confirmed through qRT-PCR and western blotting (Fig 4A and 4B). Compared to its negative controls, these miR-218 overexpressing U87 and U251 stable cell lines demonstrated a significant decrease in their expression of HK2 mRNA and protein levels, as confirmed by qRT-PCR and western blotting (Fig 4C and 4D).

Further, luciferase reporter assay was employed to assess the miR-218 mediated post-transcriptional alteration of HK2. The result showed no difference in the luciferase activity between the pmirGLO-HK2 3’-UTR transfected, pmirGLO-HK2 3’-UTR-mut transfected and negative control cells, indicating that HK2 is not the direct target of miR-218 (Fig 4E). Scale bar = 100 μm. Error bars represent SEM, *, P<0.05; **, P<0.01; ***, P<0.001.

### Bmi1 may facilitate the miR218 mediated HK2 regulation

Considering our previously established miR-218/Bmi1 pathway in glioma inhibition, we hypothesized that Bmi1 may fill the gap between miR-218 induced HK2 suppression. To assess the hypothesis, Bmi1 suppressed stable U87 and U251 cells were successfully obtained (Fig 4F), and were subject to qRT-PCR and western blot analysis. In accordance with our expectation, knockdown of Bmi1 expression in glioma cells dramatically decreased their expression of both HK2 mRNA and protein level (Fig 4G and 4H). This result was also consistent with the effect of miR-218 overexpression on HK2 expression, which further supports the hypothesis that Bmi1 may be the key factor of miR-218 mediated HK2 regulation.

### The effects of overexpression of miR-218 and knockdown of Bmi1 on AKT/pAKT and HIF-1α

To further assess the contribution of miR-218 and Bmi1 on glioma development, we examined the expression levels of pAKT and HIF-1α, which belong to the PI3K pathway. As shown in S1 Fig, there were increased expressions of both pAKT and HIF-1α proteins after overexpression of miR-218 and knockdown of Bmi1. Although these results were contrary to our expectation, further supported that miR-218 might regulate glioma cells development by regulating many genes besides HK2.

## Discussion

Though aerobic glycolysis seems to be an inefficient metabolic pathway in terms of ATP production, in the presence of adequate nutrition the pathway demonstrates efficient biomass production, necessary for cell division, while maintaining a viable ATP/ADP ratio [7]. This switch in metabolic pathway can support cellular proliferation and increase invasive ability, suppressing mitochondria mediated apoptosis, and thus is a concern in cancerous condition and needs to be addressed [8,9,23]. In these regards, HK2, one of the prime driver of the Warburg Effect in cancer cells, is of special interest lately.

Consistent with previous reports, the results of our study reveal an increase in the HK2 expression in glioma condition, as confirmed through glioma cell lines and human glioma tissue analysis [15,24]. This increase in HK2 expression was unrelated to age and gender of the assessed patients, but was positively correlated with tumor stage. This suggests an increase in glycolytic activity at higher grade glioma, which may correspond to its aggressive features. Further, the shRNA-targeted reduction of HK2 expression in glioma cell lines decreased their proliferative, invasive and migrating abilities. Moreover, xenograft tumors derived from such HK2 silenced U87 cells were reduced in weight and volume, compared to those formed by negative control cells. These data suggest HK2 as an oncogene, and may play a central role in the pathogenesis and progression of glioma. The oncogenic role of HK2 has been observed in other cancer conditions too, including gastric cancer [25], hepatocellular carcinoma [26–27], renal cell carcinoma [28], and in brain metastases of breast cancer [29–30]. Further, a negative correlation was established between patient survival and HK2 expression in hepatocellular carcinoma [27] and brain metastases of breast cancer [30]. Also, in agreement with our findings, some studies have shown that decreased HK2 expression resulted in decreased cell proliferation, increased apoptosis, and diminished tumor growth in glioblastoma [15], colon cancer [31], thyroid cancer [32] and hepatocellular carcinoma [33]. Such results support the need for investigating HK2 inhibitors as potential treatment for the associated cancer, including the glioma condition.

There are several mechanisms involved in regulation of HK2 expression. Both glucose phosphorylation and increased localization of HK2 to the mitochondrial membrane are reported to contribute to increased HK2 expression [34]. Further, hypoxia induced activation and stabilization of HIF-1α transcription factor promotes the expression of HK2 [35]. Akt/mTORC1 pathway is also shown to regulate HK2 expression [36]. Silencing HK2 expression exerted profound effects on aerobic glycolysis, indicating the importance of HK2 in achieving maximal glycolysis [37]. Pharmaceutically or genetically intervened inhibition of HK2 results in decreased tumor cell’ aggressiveness while rendering them susceptible to chemo/radio therapy [15,38]. This indicates the importance of maximal glycolysis, and thus HK2, in tumor survival and progression. But, the exact mechanism involved in HK2-mediated tumor progression, however, remains largely unexplored.

The microRNAs, with their ability to target multiple genes, play an important role in many cancer processes, and is thus a hot spot of research in the field [39–41]. Several microRNAs are shown to suppress tumor by regulating their glycolytic pathway [30,42–45]. miR-218 was one such microRNA which was drastically downregulated in human glioma compared to normal brain tissues [46–48]. It has been shown that miR-218 regulates glioma cell invasion by downregulating IkB kinase-b and LEF1 [49]. Likewise, previously we have shown that restoring the expression of miR-218 regulated a broad range of genes involved in glioma cell development, with Bmi1 being defined as its direct functional target, and dramatically reduced the migration, invasion, proliferation, and self-renewal of glioma cells [22]. Among the affected genes, HK2 was also found downregulated, and was listed under the targets of miR-218, with bioinformatic software. In the present study, it was also shown that the inhibition of HK2 resulted in a reduction of proliferation, migration and invasion of glioma cells, an effect that resembled the effect of miR-218 overexpression. Thus, HK2 as a potential target of miR-218 was the focus for further functional analysis.

Indeed, it was observed that overexpression of miR-218 in glioma tumor cells did obviously decrease HK2 mRNA and protein expression, though luciferase activity analysis declined HK2 as a direct target of miR-218. Further, looking for the mediator facilitating the miR-218 induced HK2 downregulation, a positive correlation between Bmi1 and HK2 expression was established. Our previously established fact that Bmi1 is a direct functional target of miR-218 in glioma condition validates the miR-218 induced HK2 suppression. This indicates that the miR-218/HK2 axis, in addition to regulating glucose metabolism, plays an important role in controlling tumorigenesis in glioma, thus adding a novel molecular link between tumor biology and tumor metabolism.

Numerous studies have demonstrated that activation of the PI3K/Akt signaling pathway is essential to the development and/or progression of most cancer types and associated with nearly all aspects of the malignant phenotype of cancer, such as uncontrolled proliferation, resistance to cell death, invasiveness, angiogenesis and metastasis [50–51]. The phosphorylation of Akt activates downstream target genes involved in survival, proliferation, cell cycle progression, growth and migration of tumor cells, as well as angiogenesis [50–52]. pAKT and HIF-1α are involved in the PI3K pathway, and we provided evidence that these proteins were upregulated in both overexpression of miR-218 and knockdown of Bmi1 glioma cell lines. These results showed that miR-218 might regulate the PI3K pathway to influence glioma development and uncovered a novel mechanism for constitutive PI3K/Akt activation in gliomas. The other pleiotropic functions of miR-218 in glioma development require further research.

This study has a few limitations. The effect of HK2 downregulation, both shRNA and miR-218 induced, on cellular metabolism (glycolytic activity), ATP synthesis, or FDG uptake of glioma cells was not addressed. “If HK2 is a direct functional downstream target of Bmi1” was not assessed, though a direct correlation was established between Bmi1 and HK2 expression in glioma cells. Despite these limitations, this study supports/demonstrates the tumorigenic activity of HK2 in glioma condition, while also establishes a novel miR-218/Bmi1/HK2 axis in regulating the same.

In conclusion, downregulation of HK2 expression via shRNA led to the inhibition of glioma. Although further efforts are warranted for experimental confirmation, the findings highlight the importance of miR-218 in regulating HK2. These data hopefully add to the current understanding of the miR-218’s regulatory network in glioma condition, and may provide potential targets in developing glioma therapies.

## Supporting information

S1 Table The IHC scores of glioma tissues and non-neoplastic brain tissue. IRS revealed a significant increase in HK2 expression in glioma samples compared to non-neoplastic brain tissue. Further, amongst the glioma samples, HK2 expression increased significantly with the progression in tumor grade, thus establishing a positive correlation.
(DOCX)

S2 Table The exact value of the mean volume of the xenograft tumors. The mean volumes of xenograft tumors generated from HK2-silenced U87 cells were significantly smaller than those originating from its negative control cells.
(DOCX)

S1 Fig Both pAKT and HIF-1α showed increased expression after overexpression of miR-218 and knockdown of Bmi1. (A). Overexpression of miR-218 up-regulated the protein level of pAKT of glioma cell lines. (B). Overexpression of miR-218 up-regulated the protein level of HIF-1α of glioma cell lines. (C). Knockdown of Bmi1 increased the pAKT protein expression. (D). Knockdown of Bmi1 increased the HIF-1α protein expression.
(TIF)

S1 File NC3Rs ARRIVE guidelines checklist. Reporting tumorigenic assay: our aim was to evaluate the tumorigenic role of HK2 through silencing the HK2 expression of glioma cells and evaluating its tumor forming potential. At 20 days post-implantation, and from then on, the mean volumes of xenograft tumors generated from HK2-silenced U87 cells were significantly smaller than those originating from its negative control cells.
(DOCX)
