# Supplementary material for: Hexokinase 2 (HK2), the tumor promoter in glioma, is downregulated by miR-218/Bmi1 pathway
Source: PLoS One. 2017 Dec 8;12(12):e0189353. doi: 10.1371/journal.pone.0189353 (PMC5722312; doi:10.1371/journal.pone.0189353)
Supplement: S1 Table — IRS revealed a significant increase in HK2 expression in glioma samples compared to non-neoplastic brain tissue. Further, amongst the glioma samples, HK2 expression increased significantly with the progression in tumor grade, thus establishing a positive correlation. (DOCX) [file pone.0189353.s001.docx]

S1 Table The IHC scores of glioma tissues and non-neoplastic brain tissue

|  | Non-neoplastic brain tissue | WHO I glioma | WHO II glioma | WHO III glioma | WHO IV glioma |
| --- | --- | --- | --- | --- | --- |
| Immunoreactivity score of HK2 | 1±0.2 | 2.2±0.7 | 4.2±0.6 | 6.3±1.3 | 8.9±1.1 |
