# Supplementary material for: Hexokinase 2 (HK2), the tumor promoter in glioma, is downregulated by miR-218/Bmi1 pathway
Source: PLoS One. 2017 Dec 8;12(12):e0189353. doi: 10.1371/journal.pone.0189353 (PMC5722312; doi:10.1371/journal.pone.0189353)
Supplement: S2 Table — The mean volumes of xenograft tumors generated from HK2-silenced U87 cells were significantly smaller than those originating from its negative control cells. (DOCX) [file pone.0189353.s002.docx]

S2 Table The exact value of the mean volume of the xenograft tumors

| Day | HK2 shRNA | NC |
| --- | --- | --- |
| 1 | 0 | 11.66 |
| 3 | 1.6 | 80.7 |
| 5 | 12.08 | 112.7 |
| 7 | 15.82 | 143.5 |
| 9 | 34.5 | 309.4 |
| 12 | 38.5 | 504.04 |
| 14 | 133.1 | 1061.52 |
| 17 | 385.42 | 1680.2 |
| 20 | 1576.196 | 4157.32 |
| 22 | 1778.84 | 4727.7 |
| 24 | 1948.4 | 5932.2 |
| 26 | 2364.22 | 8324.22 |
| 28 | 2388.24 | 9467.2 |

P=0.011
