# Supplementary figures and images for: Hexokinase 2 (HK2), the tumor promoter in glioma, is downregulated by miR-218/Bmi1 pathway
Source: PLoS One. 2017 Dec 8;12(12):e0189353. doi: 10.1371/journal.pone.0189353 (PMC5722312; doi:10.1371/journal.pone.0189353)

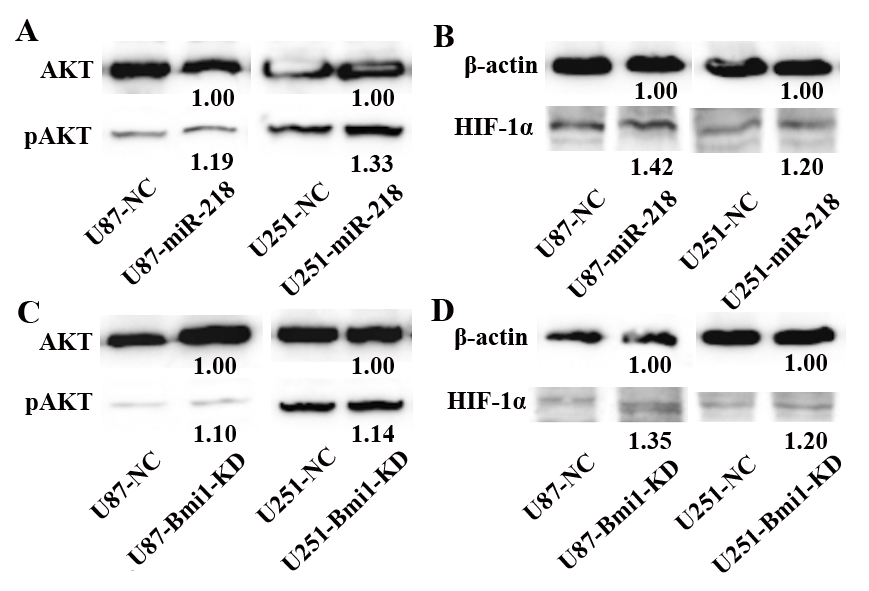

Supplement: S1 Fig — (A). Overexpression of miR-218 up-regulated the protein level of pAKT of glioma cell lines. (B). Overexpression of miR-218 up-regulated the protein level of HIF-1α of glioma cell lines. (C). Knockdown of Bmi1 increased the pAKT protein expression. (D). Knockdown of Bmi1 increased the HIF-1α protein expression. (TIF) [file pone.0189353.s003.tif]
