# Supplementary material for: Hexokinase 2 (HK2), the tumor promoter in glioma, is downregulated by miR-218/Bmi1 pathway
Source: PLoS One. 2017 Dec 8;12(12):e0189353. doi: 10.1371/journal.pone.0189353 (PMC5722312; doi:10.1371/journal.pone.0189353)
Supplement: S1 File — Reporting tumorigenic assay: our aim was to evaluate the tumorigenic role of HK2 through silencing the HK2 expression of glioma cells and evaluating its tumor forming potential. At 20 days post-implantation, and from then on, the mean volumes of xenograft tumors generated from HK2-silenced U87 cells were significantly smaller than those originating from its negative control cells. (DOCX) [file pone.0189353.s004.docx]

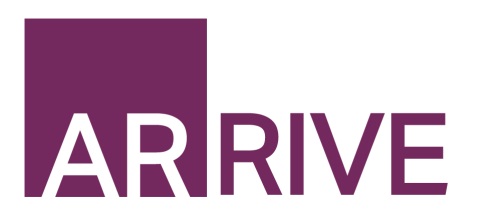


The ARRIVE Guidelines Checklist

Animal Research: Reporting In Vivo Experiments

Carol Kilkenny^1^, William J Browne^2^, Innes C Cuthill^3^, Michael Emerson^4^ and Douglas G Altman^5^

*^1^The National Centre for the Replacement, Refinement and Reduction of Animals in Research, London, UK, ^2^School of Veterinary Science, University of Bristol, Bristol, UK, ^3^School of Biological Sciences, University of Bristol, Bristol, UK, ^4^National Heart and Lung Institute, Imperial College London, UK, ^5^Centre for Statistics in Medicine, University of Oxford, Oxford, UK.*

|  | | ITEM | RECOMMENDATION | Section/ Paragraph |
| --- | --- | --- | --- | --- |
| 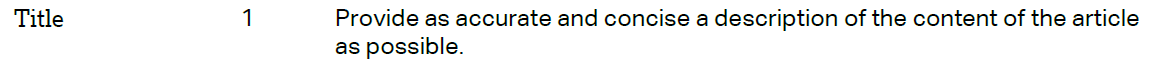 | | | Hexokinase 2 (HK2), the tumor promoter in glioma, is downregulated by miR-218/Bmi1 pathway |  |
| 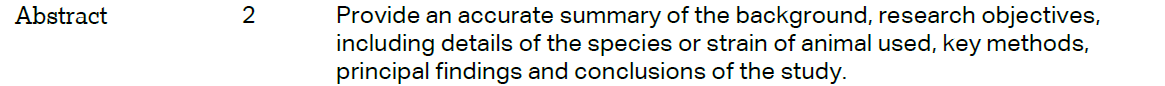 | | | BACKGROUND AND PURPOSE: HK2 is crucial for the Warburg Effect of human glioma. Our aim was to evaluate the tumorigenic role of HK2 through silencing the HK2 expression of glioma cells and evaluating its tumor forming potential.  EXPERIMENTAL APPROACH: HK2-silenced U87 stable cells and their corresponding negative controls were implanted in the left and right flanks, respectively, of 4-week-old female NOD/SCID mice (3.0×10^6^/200 mL per mice, 6 mice per cell line). Starting from day 1, the next day after cell implantation, tumor volumes were determined by measuring its length (a) and width (b) every other day, up to 28 days. The tumor volume (V) was calculated according to the formula V=(ab)^2^/2.  KEY RESULTS: At 20 days post-implantation, and from then on, the mean volumes of xenograft tumors generated from HK2-silenced U87 cells were significantly smaller than those originating from its negative control cells  CONCLUSIONS AND IMPLICATIONS: Our findings confirmed the tumorigenic activity of HK2 in glioma. |  |
| INTRODUCTION | | |  |  |
| 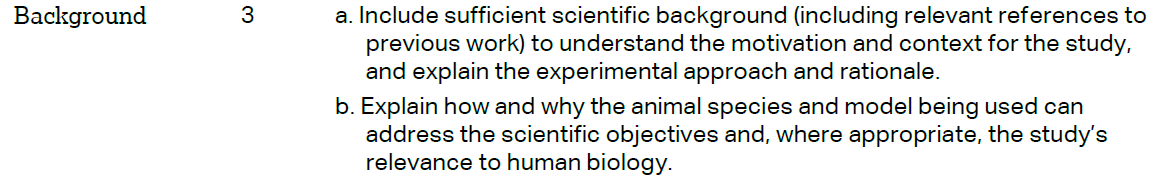 | | | Glioma was the most common primary tumor of the central nervous system, which was characterized by aggressive proliferation, migration and invasion abilities. The median survival of most glioma patients was as low as one to two years, with the 5 year survival rate staying below 10% [1], [2]. Angiogenesis was one of the hallmark malignancy parameter of glioma. Aerobic glycolysis, a unique metabolic phenomenon that convert glucose into lactic acid even in the presence of oxygen, referred to as the Warburg Effect, was noted in most solid tumors [3], [4], [5]. When compared to normal cells, tumor cells preferentially utilize this far less efficient process for ATP production, which also increased the cell’s proliferation, invasiveness and apoptosis resistance [6], [7]. This high rate of glycolysis in tumor cells, including gioma cells, was presumably ascribed to up-regulation of key catalytic enzymes in glycolysis, especially hexokinases, more specifically HK2 [8], [9]. Indeed, elevated levels of HK2 had been found in many human tumors, localized to the outer membrane of mitochondria by binding the voltage-dependent anion channel (VDAC) transporter and thus had preferential access to mitochondrial ATP [10], [11]. In addition to its critical metabolic role, HK2 could also promote glioma survival, against chemo or radiation insult, by repressing mitochondria mediated apoptotic pathway in glioma cells [12]. Thus, HK2 was of great interest in recent years and efforts were being made in understanding the associated underlying molecular mechanisms in glioma, towards which the present work was also dedicated.  To evaluate the role of HK2 in the proliferation of glioma U87 cells *in vivo*, we selected NOD/SCID mice. These animals are valuable to research because they can receive many different types of tumor grafts, as they mount no rejection response. These xenografts are commonly used in research to test new methods of treating tumors. |  |
| 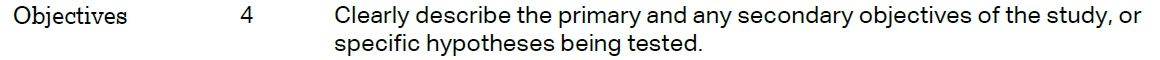 | | | The objectives of this study were to determine the contribution of HK2 in the proliferation of glioma cells *in vivo*. |  |
| METHODS | | |  |  |
| 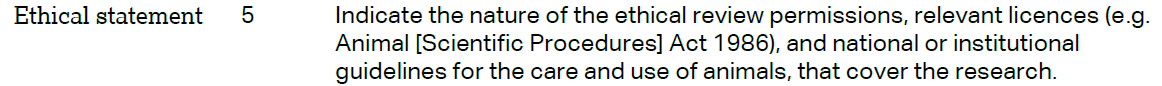 | | | All animal experiments were approved by the Research Ethics Committee of Tangdu Hospital for the care and use of animals. |  |
| 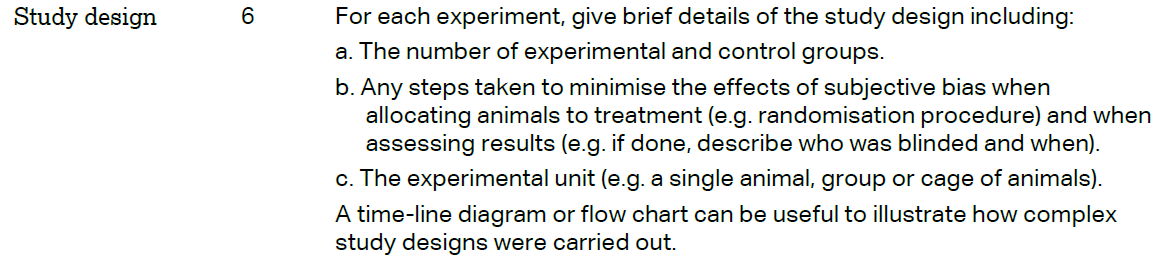 | | | Two groups of 6 mice each were studied: A: HK2-silenced U87 cells were implanted in the left flanks; B: The negative control U87 cells were implanted in the right flanks.  Mice were randomized into two different groups by picking numbers out of a hat.  In the study, each mouse was the experimental unit.  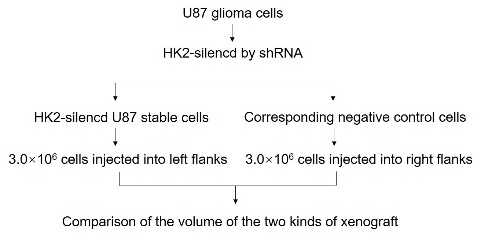 |  |
| 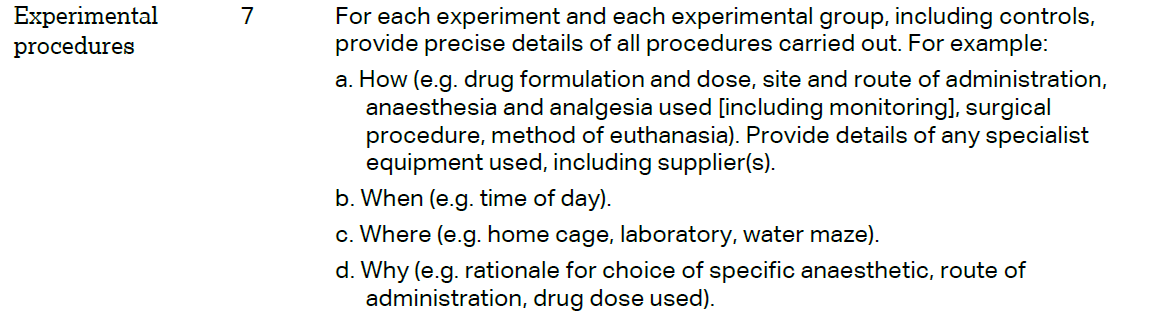 | | | HK2-silenced U87 stable cells and their corresponding negative controls were implanted in the left and right flanks, respectively, of 4-week-old female NOD/SCID mice, which were purchased from Animal Center of Fourth Military Medical University. 3.0×10^6^ of two kinds of glioma cells were suspended in 200 ml of PBS, then injected into the left and right flanks, respectively. Starting from day 1, the next day after cell implantation, tumor volumes were determined by measuring its length (a) and width (b) every other day, up to 28 days. The tumor volume (V) was calculated according to the formula V=(ab)^2^/2.  All the experiments were conducted in the light phase.  All the experiments were conducted in the laboratory.  According to our previous studies, 3.0×10^6^ glioma cells were minimally required for the forming of tumor. More cells needed more PBS to suspended for the injection, it was more harmful for the mice. |  |
| 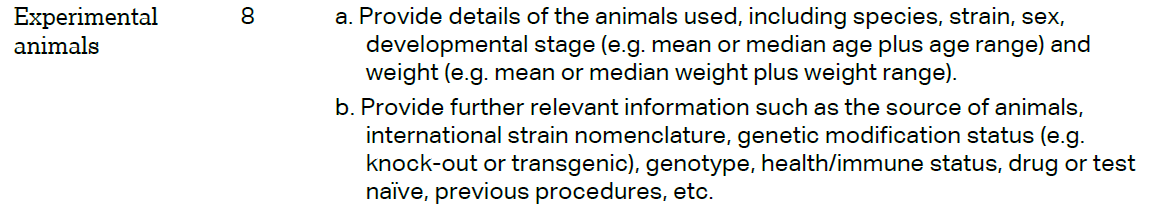 | | | Female NOD/SCID mice (20.0 ± 2.0 g), aged 4 weeks, were included (n=6).  Six NOD/SCID mice (Animal Center of Fourth Military Medical University) were obtained and acclimatized for at least 48 h. Vendor health reports indicated that the mice were free of known viral, bacterial and parasitic pathogens. |  |

The ARRIVE guidelines. Originally published in *PLoS Biology*, June 2010^1^

| 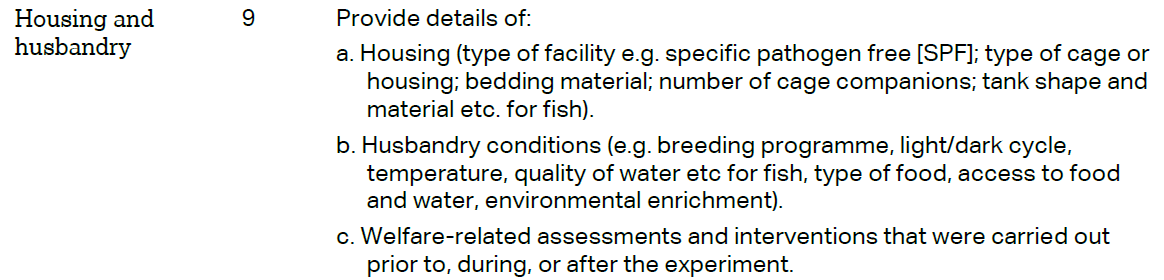 | Animals were housed with an inverse 12 hours day-night cycle with lights on at 8:30pm in a temperature (22 ± 1 ℃) and humidity (55 ± 5%) controlled room. Prior to injection the animals were housed in cages filled with hygiene animal bedding.  All mice were allowed free access to water and a maintenance diet (obtained from Animal Center of Fourth Military Medical University) in a 12-hour light/dark cycle, with room temperature at 21 ± 2 ℃. All cages contained wood shavings.  During the post-injection period, all the mice were under the tender care to relieve the pain. | |
| --- | --- | --- |
| 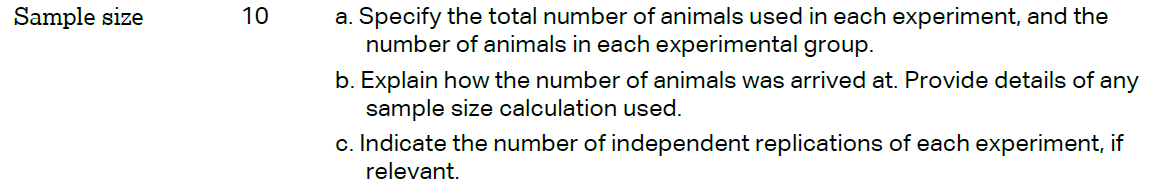 | Six healthy mice were divided into two groups of six each. HK2-silenced U87 stable cells and their corresponding negative controls were implanted in the left and right flanks, respectively.  For animal experiments, sample size, no less than 6, can basically eliminate individual differences. More samples make a little sense.  The experiment was repeated six times. | |
| 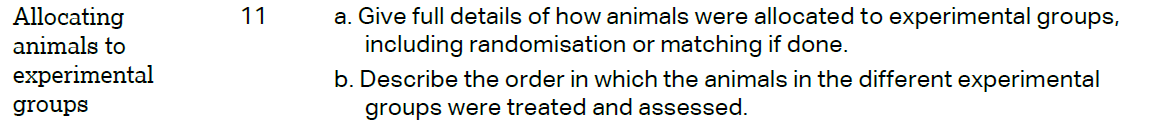 | All the mice were firstly numbered in ascending order, then were randomized into two different groups by picking numbers out of a hat.  All the mice were injected in ascending order. | |
| 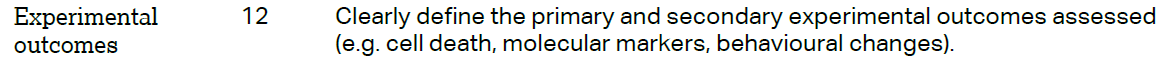 | At 20 days post-implantation, and from then on, the mean volumes of xenograft tumors generated from HK2-silenced U87 cells were significantly smaller than those originating from its negative control cells (p=0.011). | |
| 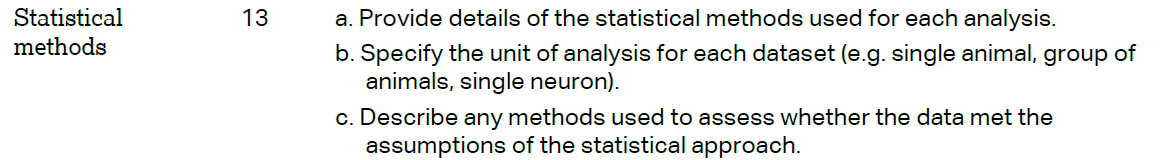 | Data were expressed as the means ± standard error mean (SEM) from three independent experiments. Two independent sample t-tests were performed using GraphPad Prism 5.0 software in order to detect significant differences in measured variables among groups. P value < 0.05 was considered to be statistically significant.  The experimental unit was an individual animal.  Test for normality was performed by Kolmogorov-Smirnov test. | |
| RESULTS |  | |
| 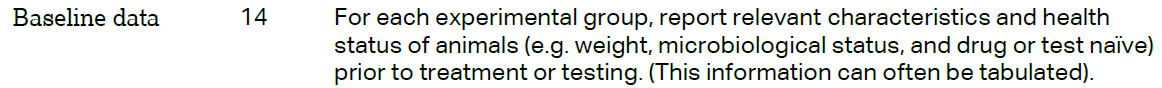 | The animal’s health status was monitored throughout the experiments and the mice were free of all viral, bacteria, and parasitic pathogens according to Animal Center of Fourth Military Medical University guidelines. | |
| 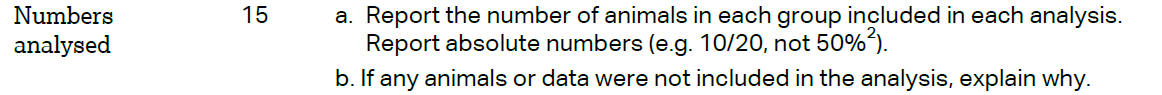 | Xenografts formed in both flanks of all the six mice, with a positive rate of 100% (6/6). | |
| 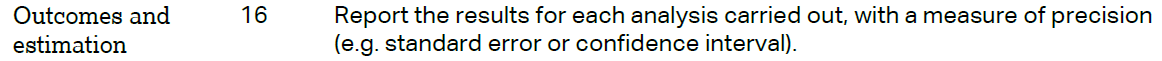 | Table 1 The exact value of the mean volume of the xenograft tumors   \| Day \| HK2 shRNA \| NC \| \| --- \| --- \| --- \| \| 1 \| 0 \| 11.66 \| \| 3 \| 1.6 \| 80.7 \| \| 5 \| 12.08 \| 112.7 \| \| 7 \| 15.82 \| 143.5 \| \| 9 \| 34.5 \| 309.4 \| \| 12 \| 38.5 \| 504.04 \| \| 14 \| 133.1 \| 1061.52 \| \| 17 \| 385.42 \| 1680.2 \| \| 20 \| 1576.196 \| 4157.32 \| \| 22 \| 1778.84 \| 4727.7 \| \| 24 \| 1948.4 \| 5932.2 \| \| 26 \| 2364.22 \| 8324.22 \| \| 28 \| 2388.24 \| 9467.2 \|   P=0.011 | |
| 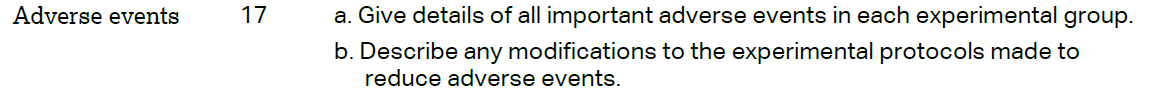 | No adverse events occurred in both experimental groups. | |
| DISCUSSION |  | |
| 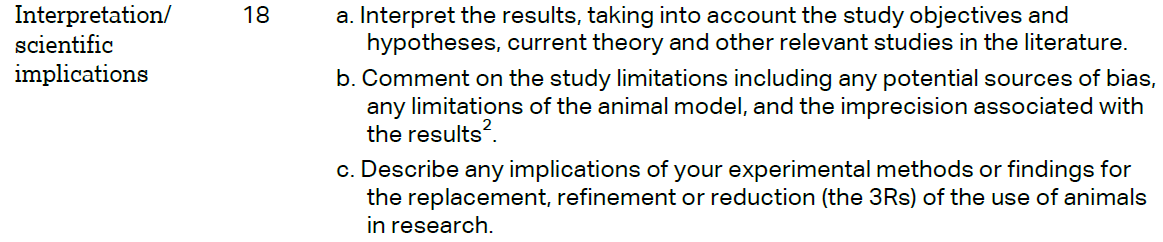 | Consistent with previous reports, the results of our study reveal an important role of HK2 in glioma. The shRNA-targeted reduction of HK2 expression in glioma cell lines decreased their proliferative, invasive and migrating abilities. Moreover, xenograft tumors derived from such HK2 silenced U87 cells were reduced in weight and volume, compared to those formed by negative control cells. These data suggest HK2 as an oncogene, and may play a central role in the pathogenesis and progression of glioma.  A limitation of this study is the fact that we didn’t establish orthotopic implantation model of human glioma in mice to better explore the tumorigenic role of HK2 in glioma forming *in vivo*.  The application of xenograft makes it possible for researchers to detect the forming of tumors *in vivo* and evaluate the effects of kinds of genes and drugs for cancer therapy. Moreover, it reduces the use of other kinds of animals in research. | |
| 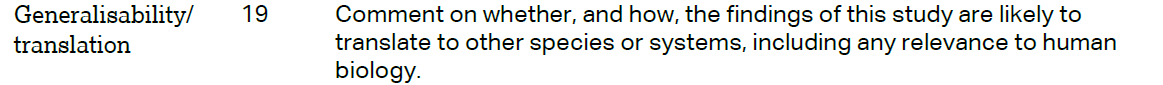 | Downregulation of HK2 expression via shRNA led to the inhibition of glioma and the findings highlighted the importance of HK2 in the proliferation of glioma. These data hopefully add to the current understanding of HK2 in glioma condition, and provide potential targets in developing glioma therapies. | |
| 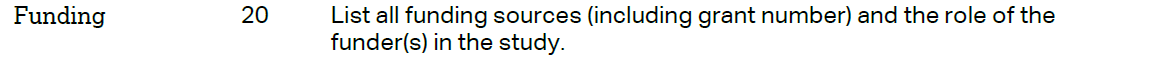 | | National Natural Science Foundation of China, Award Number: 81272419;  National Natural Science Foundation of China, Award Number: 81572983, Recipient: Yanyang Tu: Supervision, Writing: review & editing;  Social Development of Technology Research Projects in Shaanxi Province, Award Number: 2016SF191, Recipient: Hui Liu: Methodology, Writing: original draft;  Social Development of Technology Research Projects in Shaanxi Province, Award Number: 2015SF027, Recipient: Pengxing Zhang: Methodology. |


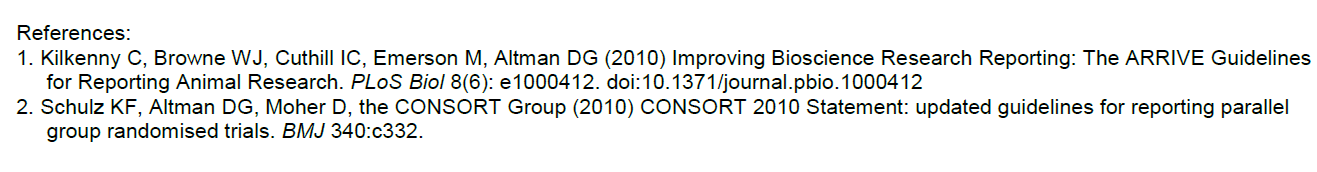

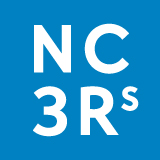


**References**

1. Davis FG, McCarthy BJ, 2001. Current epidemiological trends and surveillance issues in brain tumors. *Expert Rev Anticancer Ther*, 1: 395-401.

2. Weller M, Cloughesy T, Perry JR, et al., 2013. Standards of care for treatment of recurrent glioblastoma--are we there yet? *Neuro oncology*, 15: 4-27.

3. Warburg, O, 1956. On the origin of cancer cells. *Science*, 123: 309-314.

4. Mathupala SP, Ko YH, Pedersen PL, 2009. Hexokinase-2 bound to mitochondria: cancer’s stygian link to the “Warburg Effect” and a pivotal target for effective therapy. *Semin in Cancer Biol*, 19: 17-24.

5. Vander Heiden MG, Cantley LC, Thompson CB, 2009. Understanding the Warburg effect: the metabolic requirements of cell proliferation. *Science*, 324: 1029-1033.

6. Stern R, Shuster S, Neudecker BA, et al, 2002. Lactate stimulates fibroblast expression of hyaluronan and CD44: the Warburg effect revisited. *Experimental cell research*, 276: 24-31.

7. Plas DR, Thompson CB, 2002. Cell metabolism in the regulation of programmed cell death. *Trends in endocrinology and metabolism: TEM*, 13: 75-78.

8. Mathupala SP, Ko YH, Pedersen, PL, 2006. Hexokinase 2: cancer’s double-edged sword acting as both facilitator and gatekeeper of malignancy when bound to mitochondria. *Oncogene*, 25: 4777-4786.

9. Wolf A, Agnihotri S, Munoz D, et al, 2011. Developmental profile and regulation of the glycolytic enzyme hexokinase 2 in normal brain and glioblastoma multiforme. *Neurobiology of disease*, 44: 84-91.

10. Pedersen PL, 2007. Warburg, me and Hexokinase 2: Multiple discoveries of key molecular events underlying one of cancers’ most common phenotypes, the “Warburg Effect”, elevated glycolysis in the presence of oxygen. *J Bioenerg Biomember*, 39: 211-222.

11. Pedersen PL, 2008. Voltage dependent anion channel (VDACs): a brief introduction with a focus on the outer mitochondrial compartment’s roles together with hexokinase-2 in the “Warburg effect” in cancer. *J Bioenerg Biomember*, 40: 123-126.

12. Wolf A, Agnihotri S, Micallef J, et al, 2011. Hexokinase 2 is a key mediator of aerobic glycolysis and promotes tumor growth in human glioblastoma multiforme. *J Exp Med*, 208: 313-326.
